# Long term clinical outcome after success re-attempt percutaneous coronary intervention of chronic total occlusion

**DOI:** 10.1186/s12872-023-03045-w

**Published:** 2023-01-16

**Authors:** Wenzheng Li, Zheng Wu, Tong Liu, Xiaofan Wu, Jinghua Liu

**Affiliations:** Center of Coronary Artery Disease, Beijing Anzhen Hospital, Capital Medical University, Beijing Institute of Heart, Lung and Blood Vessel Diseases, Beijing, China

**Keywords:** Chronic total occlusion, Percutaneous coronary intervention, Long-term outcomes, TVR, MACE

## Abstract

**Background:**

To evaluate the long-term outcome after re-attempt CTO-PCI.

**Methods:**

This is a retrospective cohort study that included 113 re-attempt CTO-PCI patients who were consecutively registered from January 2019 to December 2020 at Beijing Anzhen Hospital's Center of Coronary Artery Disease. All patients were divided into two groups based on procedural success or failure. The primary endpoint was major adverse cardiac events (MACE), a composite of all-cause mortality, myocardial infarction and target vessel revascularization (TVR). The secondary endpoint was angina after PCI.

**Results:**

Overall, the successful re-attempt CTO-PCI was archived in 77 patients, the failed CTO-PCI was performed in 36 patients. After a median follow-up of 21.7 months (interquartile range: 10.9–26.0), the incidence of the primary outcome was significantly lower in the success group [14.2% vs. 38.9%, adjusted hazard ratio (HR) 0.351, 95% CI 0.134–0.917, P = 0.033], mainly driven by the reduction of TVR (9.1% vs. 30.6%, adjusted HR 0.238, 95% CI: 0.078–0.72, P = 0.011). Furthermore, patients who had successful re-attempt CTO-PCI had a lower risk of angina after PCI (27.3% vs.61.1%, adjusted HR 0.357, 95% CI 0.167–0.76, P = 0.008). The risk factors of TVR in the patients with successful re-attempt CTO-PCI were stent length > 100 mm (adjusted HR 21.805, 95% CI 1.765–269.368, P = 0.016) and J-CTO score > 3(adjusted HR: 9.733, 95% CI:1.533–61.797, P = 0.016).

**Conclusions:**

For the patients with previous CTO-PCI failure, a successful re-attempt CTO-PCI was associated with significantly lower MACE, which was primarily driven by a lower TVR rate. More complex CTO lesions and longer stents were the independent predictors of TVR after successful CTO-PCI.

## Background

With the introduction of novel devices and techniques, the success rate of chronic total occlusion (CTO)-percutaneous coronary intervention (PCI) has increased considerably [1]. Interventional revascularization was frequently considered for patients with CTO lesions. However, approximately 21% of patients did not have their arteries reopened, and this group should not be neglected. Furthermore, the prognosis for these patients was previously considered poor, and it is unknown whether re-attempting CTO-PCI can enhance the clinical outcome [2, 3]. Thus, we conducted this study to determine the long-term prognosis for the patients who had re-attempt CTO-PCI.

## Methods

### Patient population

The patients who received re-attempt CTO-PCI at Center of Coronary Artery Disease, Capital medical university, Beijing Anzhen Hospital, Beijing, China, between January 2019 and December 2020 were enrolled in this retrospective cohort study. Inclusion criteria were age > 18 years old, at least one native coronary artery occlusion, anginal symptoms and myocardial viability or ischemia in the territory of the occluded artery. Exclusion criteria were the estimated duration of a total occlusion less than three months, acute myocardial infarction (MI), venous grafts as target occluded vessels, side branch occlusion (i.g. diagonal or marginal branches), and life expectancy < 1 year. The optimal medical therapy was provided to all patients. All CTO-PCI procedures were performed by high-volume operators with significant experience in CTO-PCI. The study protocol fulfilled the ethical guidelines of the 1975 Declaration of Helsinki. This study was approved by the institutional ethics committee of Beijing Anzhen Hospital.

Patients were categorized into two groups according to procedural results: (1) success group, (2) failure group. The clinical data were gathered retrospectively through a review of hospital records by physicians. J-CTO (Multicenter CTO Registry in Japan) score and procedural data were retrospectively evaluated by experienced physicians. Clinical follow-up after discharge was frequently performed by telephone interviews or outpatient visits. Follow-up visits were set up every three months for the first year following discharge. Follow-up visits were arranged every 6 months for more than a year following discharge. During the follow-up visit, data on angina, mortality and MI, as well as target vessel revascularization (TVR) were obtained.

### Definitions and endpoints

A CTO was defined as a total obstruction of a native coronary artery with thrombolysis in myocardial infarction (TIMI) flow vessel grade 0 for a duration greater than 3 months. Calcification and bending was defined according to the definition in J-CTO score sheet [4]. Procedural success was defined as technical success without any in hospital adverse events. In hospital adverse events included all-cause death, Q-wave MI, stroke, recurrent angina requiring TVR with PCI, or coronary artery bypass grafting (CABG). Angina was defined as typical symptoms with a corresponding change in electrocardiogram or stress test. Angina after PCI was defined as ischemia induced chest discomfort, including recurrent angina, residual angina and CTO target vessel related angina. MI was defined according to the fourth universal definition of myocardial infarction [5]. Multi-vessel disease (MVD) was defined as a stenosis > 70% of the coronary lumen diameter in ≥ 2 of major epicardial arteries in vessels ≥ 2.5 mm or a left main stenosis > 50%. Complete revascularization was defined as treating all lesions with ≥ 50% stenosis in major epicardial coronary vessels at the index hospitalization. TVR was defined as any repeat recanalization (PCI or CABG) of any segment of the target treated CTO vessel. The primary endpoint was the major adverse cardiac events (MACE) and it was defined as a composite of all-cause mortality, MI and TVR. The secondary endpoint was angina after PCI.

### Statistical analysis

Continuous variables were expressed as mean ± standard deviation or median (inter quartile range, IQR) and compared using the student’s t-test or Mann–Whitney U test. Categorical variables were presented as numbers and percentages (%) and compared using Chi-square test or Fisher’s exact test. To determine event-free survival, the Cox proportional hazards model was utilized. Age, hypertension, ejection fraction less than 50%, calcification, MVD, J-CTO > 3, reference vessel diameter, retrograde approach were all taken into account when calculating adjusted hazard ratios(HR) and 95 percent confidence intervals(CI).Cox regression analysis was utilized to select covariates that were linked with an increased incidence of TVR following successful recanalization.The variables with *P* value < 0.10 on univariate analysis or clinical significance were included in the multivariate regression. A two-sided *P* value < 0.05 was considered statistically significant. SPSS 25.0 (IBM, USA) was applied for all statistical analyses.

## Results

### Baseline characteristic

**Fig. 1:**
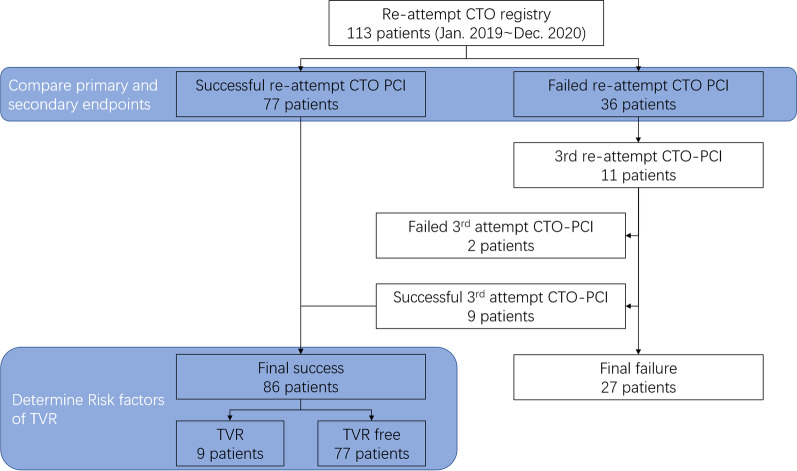
Study flowchart. CTO: chronic total occlusion; PCI: percutaneous coronary intervention; TVR: target vessel revascularization

Baseline characteristics of the study participants were reported in Table 1. Among 113 consecutive patients who underwent re-attempt CTO-PCI in our center, procedural success was obtained in 77 patients whereas the procedure failed in 36 patients (Fig. 1). Patients with successful procedure had less frequent hypertension, while no difference in age, sex, body mass index, history of diabetes, lipid disorder, chronic kidney disease, previous MI, previous CABG and smoking.

**Table 1:** Baseline and procedural characteristics

	Success (n = 77)	Failure (n = 36)	*P* value
Age	57.86 ± 10.30	59.44 ± 10.38	0.448
Male	64 (83.1%)	33 (91.7%)	0.225
BMI	26.78 ± 5.65	26.65 ± 3.91	0.898
Hypertension	51 (66.2%)	32 (88.9%)	0.011
Diabetes	30 (39.0%)	11 (30.6%)	0.387
Lipid disorder	61 (79.2%)	30 (83.3%)	0.607
CKD	6 (7.8)	4 (11.1%)	0.723
Previous MI	21 (27.2%)	13 (36.1%)	0.340
Previous CABG	2 (2.6%)	2 (5.6%)	0.591
Smoking	38 (49.4%)	17 (47.2%)	0.833
*CTO target vessel*			0.225
LAD	30 (39.0%)	15 (41.7%)	
LCX	4 (5.2%)	5 (13.9%)	
RCA	43 (55.8%)	16 (44.4%)	
MVD	50 (64.9%)	24 (66.7%)	0.927
Retrograde approach	35 (45.5%)	14 (38.9%)	0.512
EF ≤ 50%	10 (13.0%)	3 (8.3%)	0.546
Duration since last attempt ≥ 3 months	36 (46.8%)	21 (58.3%)	0.251
CTO segment length ≥ 20 mm	30 (39.0%)	21 (58.3%)	0.054
Calcification	29 (37.7%)	24 (66.7%)	0.004
Bending	20 (26.0%)	8 (22.2%)	0.667
Tapered proximal cap	34 (44.2%)	11 (30.6%)	0.169
J-CTO score > 3	16 (20.8%)	16 (44.4%)	0.009
Reference vessel diameter	2.71 ± 0.41	2.42 ± 0.55	0.002
Bifurcation	51 (66.2%)	29 (80.6%)	0.119
ISR	4 (5.2%)	3 (8.3%)	0.519
Stent implantation	76 (98.7%)	–	
Stent length > 100 mm	10 (13.0%)	–	
*Re-attempt failure reason*			
GW failure		31 (86.1%)	
Device failure		2 (5.6%)	
Complication		3 (8.3%)	

Values are mean ± standard deviation, or n(%). *BMI* body mass index; *CKD* chronic kidney disease; *MI* myocardial infarction; *CABG* coronary artery bypass graft; *CTO* chronic total occlusion; *LAD* left anterior descending; *LCX* left circumflex; *RCA* right coronary artery; *MVD* multi-vessel disease; *EF* ejection franction; *ISR* in-stent restenosis

Angiographic characteristics were presented in Table 1. Presence of calcification and J-CTO score > 3 was more frequent in failure group (66.7% vs. 37.7%, P = 0.004, 44.4% vs. 20.8%, P = 0.009, respectively). Larger reference vessel diameter was observed in failure group (2.71 ± 0.41 mm vs. 2.42 ± 0.55 mm, P = 0.002). 55 out of 77 patients (71.4%) received complete revascularization in the success group.

### Clinical outcomes and follow-up

**Fig. 2:**
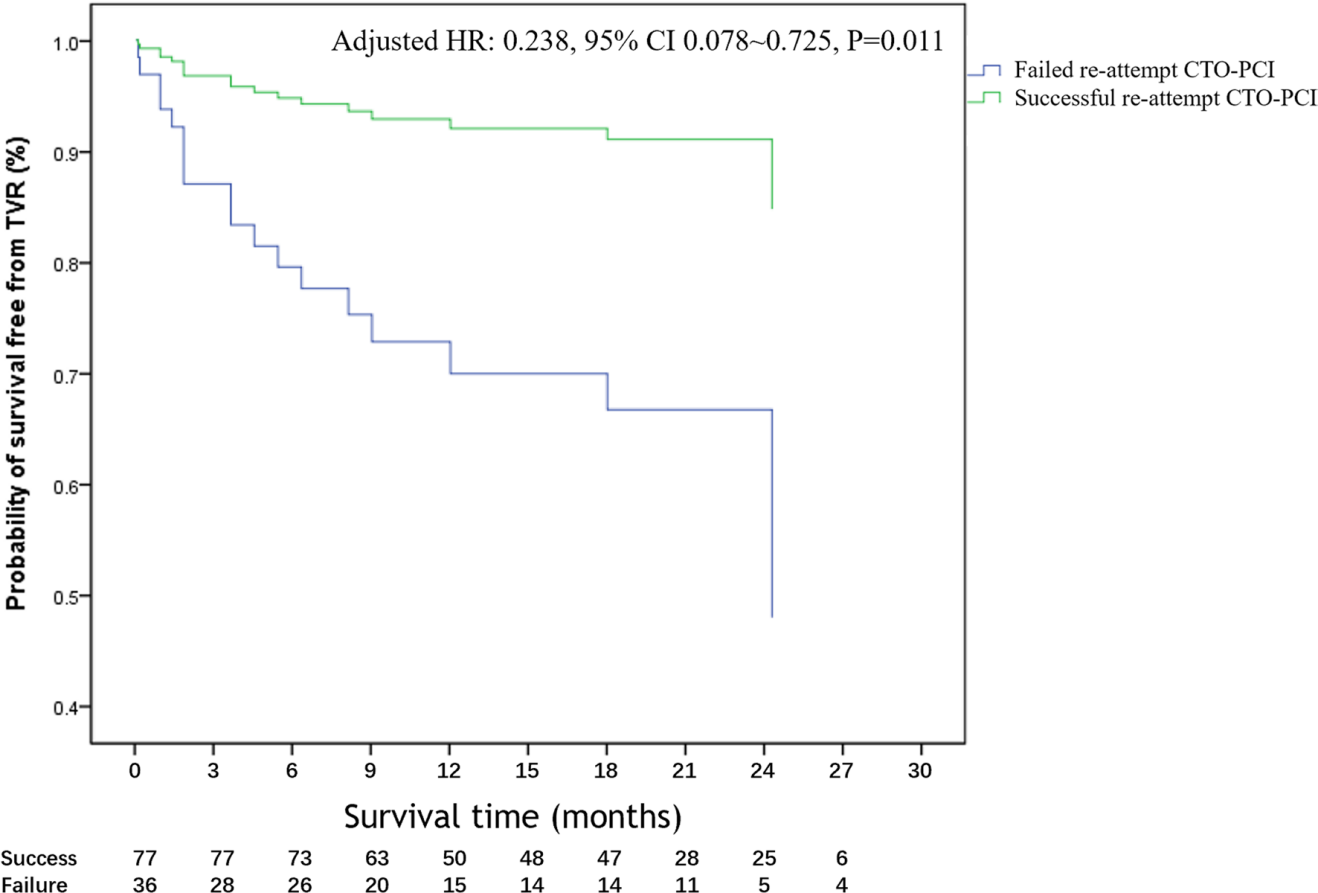
Kaplan–Meier event-free survival curve of TVR. CI: confidence intervals; CTO: chronic total occlusion; HR: hazard ratio; PCI: percutaneous coronary intervention; TVR: target vessel revascularization

Table 2 shows occurrence rates for the success and failure groups using Cox proportional hazards regression. All of the patients in this research were followed up in person or by phone. With in one year of discharge, 47.8% of patients had attended the hospital for their follow-up, and after one year, most patient had telephone follow-up. Because of the outbreak of COVID-19, other patients who did not dwell in Beijing were contacted by phone. The median follow-up time was 21.7 months (IQR 10.9–26.0). 25 patients were adjudicated to have all-cause mortality, MI or TVR. MACE were significantly lower in the success group than in the failure group (14.2% vs. 38.9%, adjusted hazard ratio (HR): 0.351, 95% CI 0.134–0.917. P = 0.033). The significant reduction of MACE in the success group was mainly driven by the improved outcome of TVR (9.1% vs. 30.6%, adjusted HR: 0.238, 95% CI 0.078–0.724, P = 0.011, Fig. 2). Subsequent PCI was underwent to achieved TVR. There was no statistically significant difference in all-cause mortality and MI in our analysis. During the follow-up period, patients who had a successful re-attempt CTO-PCI had fewer angina after PCI than those who had a failed operation (27.3% vs.61.1%, adjusted HR 0.357, 95% CI 0.167–0.762, P = 0.011).

**Table 2:** Cumulative event rates between patients with different results

	Success (n = 77)	Failure (n = 36)	Adjusted HR (95% CI)	*P* value
MACE	11 (14.2%)	14 (38.9%)	0.351 (0.134–0.917)	0.033
All-cause mortality	3 (3.9%)	5 (13.9%)	0.782 (0.113–5.400)	0.803
MI	5 (6.5%)	2 (5.6%)	5.609 (0.478–65.881)	0.170
TVR	7 (9.1%)	11 (30.6%)	0.238 (0.078–0.724)	0.011
Angina after PCI	21 (27.3%)	22 (61.1%)	0.357 (0.167–0.762)	0.008

Adjusted covariates: age, hypertension, EF ≤ 50%, MVD, calcification, J-CTO score > 3, retrograde approach and reference vessel diameter. *MACE* major adverse cardiac event; *MI* myocardial infarction; *TVR* target vessel revascularization

### Risk factor of TVR after success CTO recanalization

Eleven patients in the failure group had a third CTO-PCI attempt due to their prolonged anginal symptoms after PCI, and in some cases, patients requested (Fig. 2). Because of successful recanalization, 9 of them were reclassified to the success group. Overall, 76.1 percent of the procedures were successful. Of the 86 patients who ultimately had a successful re-attempt CTO-PCI, 9 (10.5%) had a TVR, 7 of 77 had successful recanalization on the second attempt, and 2 of 9 had successful recanalization on the third attempt. A COX regression analysis was used to determine the independent predictor of TVR after successful CTO recanalization. After adjusted covariates, the independent risk factors included J-CTO score > 3 and stent length > 100 mm were significant (Table 3; p < 0.05).

**Table 3:** Multivariate Cox regression analyses assess the risk factors of TVR in 86 cases with successful percutaneous revascularization

	Adjusted HR (95% CI)	P value
Age	0.924 (0.829–1.029)	0.151
Hypertension	1.713 (0.189–15.523)	0.632
J-CTO > 3	9.733 (1.533–61.797)	0.016
MVD	3.557 (0.388–32.592)	0.261
Retrograde approach	3.180 (0.319–31.655)	0.324
EF ≤ 50%	2.464 (0.165–36.792)	0.513
Stent length > 100 mm	21.805 (1.765–269.368)	0.016
Vessel reference diameter	0.750 (0.061–9.232)	0.822

*MVD* multi vessel disease. *EF* ejection fraction

## Discussion

The success rate of CTO-PCI and prognosis are currently unsatisfactory, particularly for re-attempt procedures. This retrospective observational study evaluated the outcomes and MACE rate after successful or failed re-attempt CTO-PCI. The main finding of this study was that successful re-attempt recanalization dramatically reduced MACE and alleviated symptoms of angina. We also demonstrated that the stent length > 100 mm and J-CTO score > 3 were risk factors for TVR following a successful re-attempt CTO-PCI.

*Angina after PCI* The symptom of angina and quality of life (QoL) can be improved by CTO-PCI [6]. The presence of angina was also an indication of percutaneous recanalization [7]. A meta-analysis encompassed 7288 patients and observed an average of 6 years of follow-up time conducted [8]. The prognosis following successful vs. failed CTO-PCI was examined in this study. Successful recanalization was associated with a significant reduction in residual/recurrent angina (OR = 0.45, 95% CI 0.30–0.67) and subsequent CABG surgery (OR = 0.22, 95% CI 0.17–0.27). Moreover, compared to non-successful PCI, a recent meta-analysis showed a significant improvement in Seattle Angina Questionnaire(SAQ)- QoL post successful PCI [9]. Borgia et al. [10] observed 302 patients with CTO-PCI, 78% of patients had a successful procedure. This trial revealed that after a median follow-up of 4 years the CTO recanalization significantly improved the angina-related QoL than before (82 vs. 53, P < 0.05). In the success group of our study, the ischemia was mitigated and the symptoms of angina were alleviated immediately after recanalization. After nearly three years of follow-up, the incidence of angina after PCI in the success group was remained statistically lower than in the failure group. We also discovered that angina after PCI was the most common reason for patients receiving the third attempt. In our study, 11 patients in the failure group underwent a third attempt. Nine of them were successfully reopened the CTO target vessel. Therefore, we argued that re-attempt CTO-PCI should still be recommended for patients who had a prior failed CTO and have clear evidence of ischemia. In patients who have had a successful re-attempt CTO-PCI, the emergence of angina pectoris is linked to incomplete revascularization, but it may also be linked to the incidence of target vessel failure.

*MACE and TVR* Failed CTO recanalization not only affected the patients’ QoL, but it also increased the risk of MACE. The long-term effects of successful CTO recanalization were the subject of debate. In most observational studies, successful CTO-PCI has been shown to improve patients' long-term MACE. A meta-analysis of 25 observational studies compared the long-term outcomes of successful versus failed CTO-PCI, and the findings revealed that successful CTO-PCI was associated with lower mortality [6]. Regardless of the quality of the collateral circulation, Jang et al. [11] observed a lower incidence of MACE after aggressive revascularization as compared with optimal medical therapy (OMT) alone. In contrast, the DECISION-CTO trial, which involved over 800 patients with CTO and was the first randomised controlled trial, came up with a negative outcome [1]. In this trial, the primary endpoint was the three-year rate of MACE (all-cause death, MI, stroke and revascularization). The incidence of MACE in PCI + OMT group was 20.6%, while OMT group was 19.6% (P = 0.008 for non-inferiority). There was also no difference in the evaluation of QoL. These results and conclusions were challenged due to delayed and incomplete patient recruitment, revascularization of other non-occlusive lesions in addition to CTO-PCI after randomization and baseline health status assessment, a high crossover rate to the PCI group (15–20%), and the inclusion of periprocedural MI in the primary outcome. Our research supports the hypothesis that CTO recanalization can improve MACE. The current study found that after a mean follow-up of 21.7 months, the incidence of MACE were significantly lower in the success group than in the failure group. MACE were reduced predominantly as a result of decreased TVR, whereas death and MI rates were comparable across the two groups.

*Risk factors of TVR* This study demonstrated that J-CTO score > 3 and stent length > 100 mm as the risk factor of TVR after successful re-attempt CTO recanalization. J-CTO score which as the earliest scoring system was frequently utilized in daily clinical practice [4], J-CTO score > 3 was considered as a very difficult lesion. Abe et al. argued that J-CTO score can predict the prognosis of patients with CTO [12]. In a five years follow-up study involving over 2000 patients, J-CTO score and residual SYNTAX score were independently associated to TVR (HR, 1.31; 95% CI: 1.11–1.54; P < 0.01) [13]. However, a small observational study enrolled only 93 patients identified that reduced TIMI flow of target vessel (OR: 11.0, 95% CI: 2.7–45.5, P = 0.001) as well as female (OR: 11.0, 95% CI: 2.1–58.5, p = 0.005) were the predictors of adverse events, indicating the predictive value of target vessel failure [14]. Surprisingly, the pre-procedural J-CTO score in our cohort did not predict the occurrence of TVR, possibly due to the small sample size. Our study found that J-CTO score > 3 was one of the risk factors for TVR. Lesions with J-CTO score of 3 or higher were complex and difficult to treat, advanced and aggressive techniques that could result in long severe dissection and intimal damage, eventually leading to TVR.

Excessive long stents related TVR may be caused in part by the negative vessel remodeling beyond the occlusion. As we know, distal vessels shrink due to insufficient perfusion pressure, and it is all too easy for the operator to misinterpret normal vessels as stenosis and select an unnecessary long stent to cover the shrunk segment [15]. Studies have shown that the perfusion pressure was restored after CTO recanalization, and the shrunk lumen was significantly increased by 15.9% from baseline to follow-up (2.06 ± 0.62 mm vs. 2.30 ± 0.55 mm, p < 0.001) [16]. Stent malposition and TVR could occur in a positive remodeling vascular bed. On the other hand, the long stent may be related to extensive dissection and hematoma which is caused by guidewire manipulation into false lumen during the intervention. The subintimal tracking and reentry (STAR) technique also require stenting of a longer coronary segment, which may lead to in-stent restenosis [17]. Deferred stenting may be a solution for reducing TVR [18, 19].

Our study had several limitations. First, selection bias is an inherent limitation in retrospective studies. Second, the sample size was not large due to the high success rate of the first CTO-PCI today and the impact of the COVID-19 pandemic on patient enrolment. Our findings should be confirmed in the future by a larger multi-center clinical trial.

## Conclusions

The group of patients who successfully re-attempt CTO recanalization had a 65% lower risk of MACE during long-term follow-up than the group of patients who failed re-attempt CTO recanalization. This association was primarily owing to a lower rate of TVR. Longer stents and more difficult CTO lesions were independent predictors of TVR after successful recanalization.

## Data Availability

The datasets used and/or analyzed during the current study are available from the corresponding author on reasonable request.

## Abbreviations

CTO: Chronic total occlusion
PCI: Percutaneous coronary intervention
MACE: Major adverse cardiac events
TVR: Target vessel revascularization
HR: Hazard ratio
CI: Confidence intervals
TIMI: Thrombolysis In Myocardial Infarction
CABG: Coronary artery bypass grafting
MI: Myocardial infarction
MVD: Multi-vessel disease
QoL: Quality of life
